# Food purchase patterns indicative of household food access insecurity, children’s dietary diversity and intake, and nutritional status using a newly developed and validated tool in the Peruvian Amazon

**DOI:** 10.1007/s12571-018-0815-2

**Published:** 2018-06-26

**Authors:** Ramya Ambikapathi, Jessica D. Rothstein, Pablo Peñataro Yori, Maribel Paredes Olortegui, Gwenyth Lee, Margaret N. Kosek, Laura E. Caulfield

**Affiliations:** 1 Department of International Health, The Johns Hopkins Bloomberg School of Public Health, Baltimore, MD USA; 2 Biomedical Investigations Unit AB PRISMA, Iquitos, Peru

**Keywords:** Food security, Validity, Peruvian Amazon, Coping strategies, Mixed methods, Indicators

## Abstract

Food security, defined as the capacity to acquire preferred food at all times, can manifest in many dimensions. Following a mixed methods approach used in India and Burkina Faso, we developed a 58-item experience-based measure in the Peruvian Amazon, based on investigator observations, relevant literature, and pre-testing with community field workers. The tool encompasses seven dimensions of food security and included measures of (1) food purchases, frequency of purchase, and location of acquisition, (2) food expenses, (3) coping mechanisms, (4) preparation of leftover food, (5) food safety (refrigerator access), (6) fishing intensity and (7) selling food. The survey was piloted among 35 randomly selected families from the Malnutrition Enteric Disease (MAL-ED) birth cohort in Santa Clara, Peru and the surrounding communities. Subsequently, based on a focus group discussion, a pile-sorting exercise, and pilot results, we reduced the survey to 36 items to be collected monthly among 203 MAL-ED households from November 2013 to January 2015. Validity and reliability were then assessed using principal component analysis and exploratory factor analysis, revealing four groups of purchase and coping strategy behaviors: (1) Sweets and sugary items, (2) Less preferred, (3) More preferred, and (4) Minimum meal. Internal consistency of the final 22-item scale had an acceptable cutoff of Cronbach’s α of 0.73. Criterion and construct validity of the factor groups revealed there were: (1) food purchase patterns that were distinctive to quality and quantity aspects of the Household Food Insecurity Access scale, (2) unique correlations of child’s intake of fats, animal source protein, fiber and other micronutrients, (3) household purchase patterns from the “more preferred” group (fish, red meat) associated with child’s weight-for-age. Food purchase and frequency, and context-specific behaviors at the household level can be used as surrogates for dietary intake patterns and nutritional status among children. Food purchase and frequency measurement is a quick, objective, non-intrusive survey method that could be used as an indicator for acute changes in household food security status with appropriate pilot testing and validation.

## Introduction

According to the 1996 World Food Summit, food security is defined as the capacity of households to have ‘physical and economic access to safe and nutritious food at all times, while still meeting dietary needs and preferences’ (Food and Agriculture Organization 1996). This widely accepted definition includes four dimensions of food security (FS): availability, access, utilization, and stability (Food and Agriculture Organization 1996; Jones et al. 2013). Grounded in qualitative research conducted in the USA, Southeast Asia and Africa, the FS construct has been operationalized into survey instruments designed for use at the household level (Chung 1997; Frongillo and Nanama 2006; Frongillo et al. 2003; Haddad et al. 1992; Maxwell et al. 1999). In the last decade, experience-based measures of FS have been recognized as requiring community-based survey tools and, therefore, benefiting from contextual tailoring. In 1992, using participatory rural appraisal methods in rural India, Chung and colleagues developed a set of FS indicators that were based on land quality, livestock ownership, readily available assets, type of crops, migration and labor (Chung 1997). More recently, the emergence of mixed-methods studies has led to a deeper understanding and quantification of the food insecurity experience. Experience-based FS tools are based on theory and aim to capture the “perceptions or experience of a household with different aspects of food insecurity as reported by a member of the household” (Pérez-Escamilla 2012). For example, Frongillo and Nanama (2006) conducted interviews with adults in peri-urban areas of Burkina Faso to create a site-specific experience-based tool, which was then used to monitor household FS (Frongillo and Nanama 2006). This tool identified a set of FS indicators: food quantity from a household ration stock, meal patterns, frequency of food purchase, psychosocial factors surrounding food insecurity, the unit of food purchase, agriculture yield, and coping strategies. More importantly, the Burkino Faso study assessed tool validity through measures of adult anthropometry and dietary intake, specifically energy intake and dietary diversity (Frongillo and Nanama 2006). Taken together, these studies promoted the approach by which qualitative research is used to determine ‘domains’ of FS, which are then operationalized into community-specific, quantitative data collection instruments, and validated using accepted indicators of nutritional status (Chung 1997; Frongillo et al. 2003; Frongillo and Nanama 2006).

In Peru, a mixed-methods study was conducted to evaluate a local adaptation of a FS and hunger module developed by the United States Department of Agriculture (Vargas and Penny 2009). More recently, Limon et al. conducted a mixed methods study in the Andean region to explore multidimensional aspects of food security (Limon et al. 2017). However, more work is needed to enhance our understanding of context-specific coping strategies and associations with dietary and nutritional outcomes in the Amazonian region. This need is particularly true for the Peruvian Amazon, where 20% of under-five children experience chronic malnutrition, and 46% are anemic (Instituto Nacional de Estadística e Informática (Perú) 2015). Amazonian communities are also undergoing a nutrition transition while livelihoods and the food economy are still driven by seasonality, geography, and river ecology, necessitating a tool capable of capturing both transitory and chronic FS experience (Chaparro and Estrada 2012; Sherman et al. 2015; 2016; Swierk and Madigosky 2014).

Following the approach of other sequential mixed-methods FS studies, we developed an experience-based FS tool for the Peruvian Amazon (Chung 1997; Frongillo 1999; Frongillo et al. 2003; Frongillo and Nanama 2006; Gittelsohn et al. 1998; Haddad et al. 1992; Maxwell et al. 1999). The aims of our paper are to 1) describe the development of a new context-specific FS tool using qualitative and quantitative methodologies; and 2) evaluate the reliability and validity of the measure with household socio-economic status and food access, as well as children’s dietary intake, diversity, and anthropometry.

## Participants and methods

### Study design and setting

This mixed-methods study was nested within the Peruvian site of the Etiology, Risk Factors, and Interactions of Enteric Infections and Malnutrition and the Consequences for Child Health and Development Project (MAL-ED) multi-site birth cohort study initiated in 2009 (MAL-ED Network Investigators 2014; Yori et al. 2014). The Peruvian site included three peri-urban communities located 15 km from the city of Iquitos in northeastern Peru, and enrolled 303 mother-child dyads over a two-year period. The MAL-ED cohort is an ideal platform to validate a new food security tool as there was synchronized collection of the household food insecurity access survey (HFIAS) (Swindale and Bilinsky 2006), a socio-economic survey, monthly quantitative 24-h dietary recalls on children, and monthly anthropometry (monthly up to 24 months, quarterly after that) (Caulfield et al. 2014; MAL-ED Network Investigators 2014). Information in the HFIAS tool was collected as part of the MAL-ED protocol to characterize FS across eight different sites in the study. Socio-economic data were also used to create a composite index (WAMI index) comprised of access to improved water source and sanitation facilities, assets, maternal education, and monthly income (Psaki et al. 2014).

**Fig. 1:**
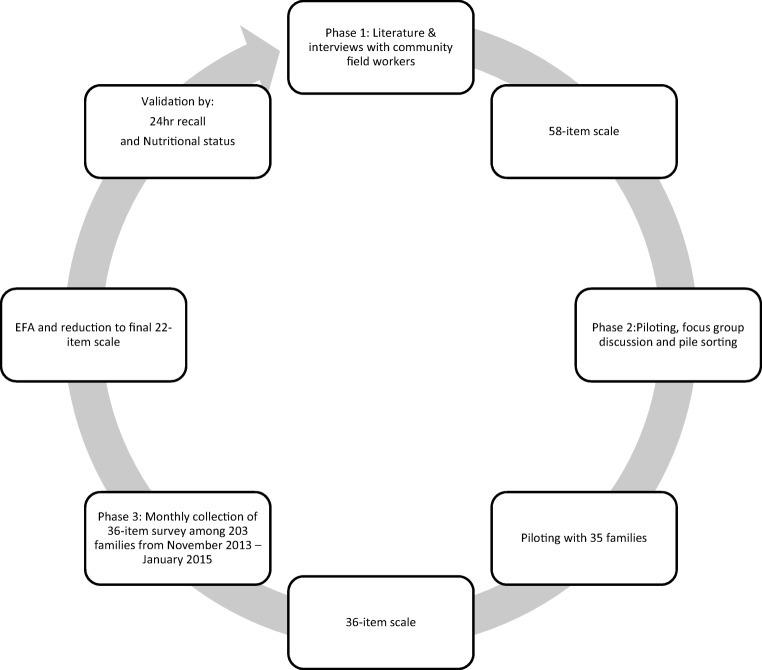
Instrument development process, conducted in three phases. Phase 1 included literature reviews and interviews with community field workers, which led to the development of 58-item scale. In phase 2, 35 pilot surveys were conducted by community field workers. Field workers were then invited to pile sorting exercises where they categorized their respective surveyed participants into five groups. Focus group discussion was conducted to ascertain the reasoning behind the placement of participants, which led to reduction of the survey to 36 items. In phase 3, monthly collection of surveys took place for 15 months. Further analyses and validation of the survey was conducted with Water and sanitation, Assets, Maternal education, Income index, HFIAS scale, dietary intake of children, and nutritional status of children

The study design was executed in three phases (Fig. 1). First, a 58-item survey tool was developed based on researcher observation in the community, interviews with community field workers (CFW), and a review of the literature, with a special focus on studies conducted in Latin America (Chung 1997; Frongillo and Nanama 2006; Gittelsohn et al. 1998; Haddad et al. 1992; Lorenzana and Sanjur 1999). Second, the 58-item survey tool was administered to 39 randomly chosen households from the MAL-ED cohort followed by pile sorting by the CFWs and focus group discussion (FGD). Third, a reduced 36-item tool was developed based on findings from phase 2, and administered to 203 households with children under five years of age from November 2013 to January 2015. Finally, EFA (Exploratory Factor Analysis) was used to reduce the 36-item tool to a final 22-item tool. The factorial, convergent and criterion validity of this tool was then evaluated in relation to WAMI index, HFIAS scale, child dietary intake, and child’s nutritional status. Caregivers provided written informed consent and protocols were approved by Institutional Review Boards from Johns Hopkins Bloomberg School of Public Health, Baltimore, MD, USA and Asociación Benéfica PRISMA, Lima, Peru.

### Phase 1: defining food insecurity measures

In developing our survey instrument, we sought to capture seven domains of FS, defined a priori (Table 1). Seven community field workers (CFWs) from the MAL-ED study who lived in the study area participated in the survey development phase, assisting primarily with content and language. Based on their input, we refined the survey instrument to include local terms and practices that are observed in households confronting food scarcity. Because our goal was to capture acute rather than chronic food insecurity, items from all domains were queries with respect to the previous week except for Domain 2, food expense items, which captured the previous day, and Domain 7, selling food items, which were queried over the previous month. The seven domains of FS are described below.

1. *Frequency of food purchase and location of acquisition:* Caregivers were asked about frequency of food purchasing and location of food acquisition because the composite of these two measures would yield an estimate of physical and economic access to food and household food flow (Frongillo et al. 2003; Gittelsohn et al. 1998). These questions related to 34 food items from five food groups, including staple foods (rice, yucca, plantains, pasta/noodles, potatoes), meat and fish (eggs, chicken, organ meat, red meat, bush meat, five types of fish and canned fish), fruits and vegetables (tomato, papaya, palm fruit, orange), snack foods (yogurt, soda, juice, low-cost crackers, high-cost crackers, cakes) and condiments (butter, cheese, palm oil, soybean oil). Based on the interviews with the CFWs, we expected the frequency combined with location to have a negative relationship with food access domains, i.e. households that frequently buy smaller quantities of food from neighborhood corner stores would be more food insecure, whereas households that purchase food items infrequently in the urban center of Iquitos, where items are generally bought in bulk, would be more likely to be food secure.
2. *Food expenses:* Two items were used to estimate food expenditure: a) total amount of money spent yesterday on food for the household, and b) the total number of household members fed yesterday (Haddad et al. 1992). Household food expenditure per capita is expected to capture the economic access aspect of FS (see Table 1), with higher per capita spending associated with greater access and consumption of animal source foods, fruits and vegetables (Melgar-Quinonez et al. 2006).
3. *Coping strategies:* Because bartering exists in this community for food and services, we asked about the frequency of bartering or borrowing in the last week, which we expected to be positively associated with food insecurity (Argumedo and Pimbert 2010; Maxwell et al. 1999; Maxwell 1996). Caregivers were also asked about additional coping strategies for food shortages, including whether the households harvested food in a garden or owned land nearby, and the types of harvested food that were consumed during the previous week. Finally, we asked if any member of the household participated in the national social program *Vaso de Leche (Glass of Milk program or VDL)* (Stifel and Alderman 2006).
4. *Preparation of leftover food:* Consuming leftover food represents one of the most common ways to access food when there is scarcity. We asked about preparation and reception of leftover food. Discussions with CFWs revealed that receiving and gifting leftover foods were common social practices (Argumedo and Pimbert 2010).
5. *Refrigerator access:* We asked about refrigerator access either through family ownership or rented use of a neighbor’s refrigerator. We expected this to be associated with food safety and higher frequency of food consumption, especially among the children.
6. *Fishing intensity:* Because the study communities are located on a river and fishing is a major economic activity, we included items about fishing intensity adapted from Ayllon’s work from the southwestern Peruvian Amazon (Ayllon 2002). In the Amazonian region, several studies have demonstrated a positive link between fishing intensity, livelihood, and consumption patterns (Ayllon 2002; Swierk and Madigosky 2014).
7. *Selling food:* We asked about selling food (cooked rice or *tamales*) and hosting *parrilladas* (charity barbeque events), as these practices were observed in the community among extremely impoverished households as a means of generating additional income. We ultimately did not include these items in the pilot survey because discussions with CFWs revealed that those events were relatively infrequent. Further, *parrilladas* may reflect one-time needs (e.g. a sick family member in need of medical treatment), rather than ongoing food insecurity experience.

**Table 1:** Domains of food insecurity practices in the Peruvian Amazon identified in Phase 1 with the hypothesized dimension of food security drawn from local and subject matter expertise and literature. Phase 2 included pile sorting and discussion of 58-item survey among 35 random households. Phase 3 involved 36-item survey conducted among 203 households from November 2013 – January 2015

Phase 1 - domains	Hypothesized dimension and component of FS (Coates et al. 2006; Leroy et al. 2015)	Examples	Phase 2	Phase 3
(1) Frequency of food purchased and location of food acquisition	Availability Physical and Economic Access Quality Quantity	• Eating less preferred staples like yucca rather than rice or noodles • Buy organ meat (chicken) compared to red meat or fresh fish • Buying bulk items at central market rather than corner stores	31 items	20 items
(2) Food expenses	Economic Access	• Spend less money on food; procure in other ways	3 items	3 items
(3) Coping strategies and food stores	Acceptability Physical access	• Bartering for services (nanny) • Borrowing on credit and having multiple credits • Participation in social programs • Consuming from own garden & types of harvested foods	12 items	5 items
(4) Preparation of leftover food	Safety and Acceptability	• Eating leftover food • Receiving and giving leftover food	6 items	3 items
(5) Food safety	Safety	• Storing food in refrigerator or renting refrigerator space with a neighbor or relative	1 item	1 item
(6) Fishing intensity	Acceptability Economic Access	• Frequency of fishing • Selling the fish	5 items	4 items
(7) Selling food	Acceptability	• Selling food to generate income • Charity BBQ sale (*parilladas*)	0 items	0 items

### Phase 2: piloting, pile sorting and focus group discussion with community field workers

The 58-item instrument was piloted among 39 randomly selected MAL-ED study households in August 2013. After the pilot survey, CFWs were invited to a pile-sorting exercise, which is a qualitative technique that relies on people to group items to examine common rationale and themes (Ryan and Bernard 2016). Out of 39 completed surveys, 35 were pile-sorted into five scaled categories (extreme food insecurity, moderate food insecurity, occasional food insecurity, food secure and could not be determined) by the CFWs based on knowledge of the household. Community field workers were familiar with household resources and practices because they had conducted twice-weekly surveillance at participants’ homes for two to three years prior. These categories were then used to compare the variability in the completed pilot survey data. After the pile sorting activity, FGD with the CFWs was held to discuss the reasoning behind their classifications of the households into the groups specified above. In addition, items deemed redundant by the CFWs were removed. After phase 2, the final tool contained 36 items (Table 1).

### Phase 3: assessing scale validity

The 36-item tool was administered monthly to monitor the FS experience among the 203 MAL-ED households actively under surveillance from November 2013 to December 2014. Subsequently, the instrument was evaluated for: (1) construct validity with (a) components of WAMI index, (b) household food insecurity access (HFIAS tool, collected every 6 months), (c) nutrient intake (24-h food recall method, collected monthly up to 36 months), (d) anthropometry (weight for age z score and length/height for age z score, depending upon whether or not the child was below 24 months of age, collected monthly); and (2) reliability (using Cronbach’s alpha). Between-subject Pearson correlation coefficients, weighted for number of repeat observations (when available) per child were used to examine the relationship between validity covariates and FS scores (generated from the exploratory factor analysis [EFA]) with statistical significance set at alpha <0.05 (Bland and Altman 1995). Data analyses were conducted using STATA Version 13.1 (StataCorp 2013).

#### Evaluation of tool validity with established FS tool and dietary intakes

Construct validity is defined as the relationship between variables that are conceptually related based on existing theories, e.g. food security status and socio-economic status (DeVellis 2016). We determined construct validity for the FS tool by examining factorial, convergent and criterion related validity. Factorial validity was evaluated through principal components analysis (PCA) with varimax rotation and polychoric correlation, followed by EFA (DeVellis 2016). PCA and EFA were performed on a subsample of data from one month (November 2013; *n* = 181). Parallel analysis with 100 repetitions was conducted to ascertain the number of factors required for the EFA. For EFA, eigenvalues >1 were included. Items were systematically deleted based on factor loadings >0.3, cross-factor loadings, homogeneity, and existing literature. As domain one included items on both food purchase and frequency, which had an innate conditional component (the number above zero for food purchase would indicate that the food was bought), we included only the frequency variable for food variables when there was over 80% consumption (for example, if 85% of the households in the community bought rice in the previous seven days, we only included the number of times they bought rice, i.e. continuous variable). We included the purchase variable (binary - yes/no) if the consumption was less than 20% across time (November 2013 to December 2014). Frequency variables were standardized. Factor scores (FAS) were generated by summarizing standardized scores by each latent factor and were used to examine convergent and criterion-related validity. For convergent validity, correlations of the FAS with the components of the WAMI index and HFIAS tool were estimated. Further, HFIAS was broken into quality, quantity and anxiety components to examine correlation with the FAS.

We explored criterion-related validity of the FAS with child’s dietary intakes and nutritional status. Because dietary recalls and anthropometric measures were collected monthly, correlation analysis was performed in the same month as the new food security survey. We evaluated intakes of energy (kcal), fat (g), animal source protein (g), meat/fish protein (g), vitamin A (μg), zinc (mg), iron (mg), sugar (g), fiber (g), dietary diversity (seven food groups), and number of food items with fish, grain, eggs, or meat, and number of desserts consumed the previous day (World Health Organization (WHO) 2010).

#### Reliability

Reliability of this instrument was assessed in two ways: 1) using Cronbach’s α for the included items (EFA) and total items in the survey subsample done in Nov 2013; and 2) Cronbach’s α of the included items from all surveys November 2013–January 2015 to establish instrument stability, since each household had 12–15 repeat measurements (DeVellis 2016).

## Results

### Phase 2: pile sorting of 58-item survey

According to pile sorting of 35 surveys, 11.4% of the households were considered extremely food insecure, 28.6% were moderately food insecure, 28.6% were occasionally food secure and 31.4% were food secure (results from the exercise are summarized in Table 2). Food insecure households tended to buy more yucca compared to food secure households, which is relatively inexpensive compared to rice. Regarding animal source protein, food insecure households tended to buy more canned tuna and organ meats compared to food secure households who bought fresh/expensive fish (e.g., *bujurqui*) and red meat.

**Table 2:** Summary of key findings on food security from the pile-sorting and focus group discussion with community field workers in the Peruvian Amazon

Pile sorting of 35 surveys	Reason for classification by community field workers
Food insecure −11.4%	• These households tend to buy more tuna and organ meat • These households ate more leftover foods • Women form these households were single, divorced or widowed • Buy food from bodega almost all of the time
Moderately food insecure – 28.6%	• These household did not have reliable breadwinner • Buy food from bodega mostly
Occasionally food insecure – 28.6%	• These households had relatives in Lima, Peru and had higher credit • These households also had more family in the community
Food secure – 31.4%	• Buy food less frequently except for rice • Buy food from center of the town/market or city • These households were less likely to eat tomato and *aguage* fruit • These households were more likely to buy juice, butter, and oil • Had higher per capita on food expenditure in the previous 24 h
Could not be determined- 0%	N/A
Other findings from pile sorting: • 90% of the households indicated that they bought at least one source of animal source protein in the last 7 days • 55% of the surveyed participants buy food on credit. Most of these (91%) are from bodega owners • 27% of the surveyed participants have credit at multiple locations to buy food. Food secure households tend to have higher credit. There was no trend observed for borrowing money to purchase food in the last seven days • The following foods showed a trend with pile sorted food security status: eggs, organ meat, red meat, *bujurqui* and *palometa* fish, and canned tuna	Other findings from the focus group discussion: • Barter markets for procuring foods for services was common, especially with women and young girls • Preference of staples consumed is indicative of food security status, with rice (more food secure) > yucca> plantains (less food secure) • Preference of meat consumed is indicative of food security status, with red meat (more food secure) > fresh fish> chicken meat > eggs> chicken organ meat> canned tuna (less food secure)

The FGD revealed that sources of income and the composition of one’s social network played a large role in how the community field workers (CFWs) pile-sorted the surveys. For example, *bodega* (corner store) owners were less willing to extend credit to families whose primary source of income came from working at the brick factory, as this source of income is unstable, especially in rainy weather. However, if households had their own business and land or had remittances coming from Lima, they were extended credit. It was also mentioned that there was less stability in household wealth from January to June, as there is a strong seasonal migration in this community due to rising river levels, which reduces access to forest products and fish (Chuquiyauri et al. 2012; Limon et al. 2017). Regarding social networks, CFWs mentioned that having relatives living in the community was indicative of the stability of food supply, because raw or prepared food along with leftovers were frequently gifted. Sometimes food was stored in neighbors’ refrigerators in exchange for caretaking or other services. In addition, the CFWs also identified marital status (e.g., widowhood) as indicative of severe food insecurity due to fewer social ties. Based on the pilot survey and FGD, we concluded that the CFWs pile-sorted food secure households because they were able to buy more meat, soda, and snacks whereas food insecure households tended to buy yucca, organ meat, eggs, canned tuna and spend less money. We removed the question regarding the VDL program because CFWs agreed that although participation is high in the program, in practice VDL is only sporadically available (Gajate and Inurritegui 2003; Gajate-Garrido 2013; Vargas and Penny 2009). We reduced the survey from 58 to 36 items; items were removed because of invariantly high response (3), no response (7), and/or redundancy (12).

### Phase 3: results from the 36-item survey from November 2013 to January 2015

Participant demographics are shown in Table 3. Overall, 203 households participated in the monthly survey across 15 months. Overall, 2769 surveys were collected from these households, and the median age of children in these households was 34 months. Mean maternal age at enrollment was 24 years and one quarter of mothers had fewer than five years of education. Average self-reported household monthly income was US$ 135. On average across the fifteen months of survey, 39.8% of the households owned their own garden, and the most common crops grown in the garden were plantains, grapefruit, yucca, coconut, papaya, and star fruit.

**Table 3:** Characteristics of the families in the Peruvian Amazon surveyed with a new food security tool at a baseline of November 2013

N	203
Visits per household	15 (14,15) ^a^
Child’s age ^b^	34 (26,41) ^a^
Food expenditure in the previous day (US$)	4.2 (3.2, 5.35)
No. ^a^ of people who were fed yesterday	5 (4, 7) ^a^
Maternal education at enrollment under 5 years (%)	24.1
Maternal age (y) at enrollment	24.2 (6.3) ^c^
Monthly household income (US$)	135 (51) ^c^
Piped water to household (%)	20.6
Pit latrine for household (%)	54
Household Food Insecurity Access Scale score, *n* = 215	3 (0, 8) ^a,d^
Weight for Age Z score, *n* = 997	−0.71 (−1.24, −0.06) ^a,d^
Length/Height for Age Z score, *n* = 997	−1.66 (−2.26, −1.21) ^a,d^
Weight for Length/Height for Age Z score, n = 997	0.47 (−0.19, 0.98) ^a,d^

^a^Results are in median (IQR)

^b^Results from the start of the survey

^c^Mean (SD)

^d^Results from all matched surveys. For example, HFIAS was collected at 18, 24, 30, and 36 months but since the starting median age at the time of first survey was 34 months, the number of matched surveys were 207 forms from 89 children. For anthropometry, there were 40 children under 24 months of age who had 2–3 repeat measures, while there were 76 children 24 months or older with 10–13 repeat measures

#### Construct validity: factorial, convergent, and criterion

##### Factorial validity

Shown in Table 4 are the results of iterative PCA and EFA on the November 2013 subsample (*n* = 181), where 36 items were reduced to 22 items based on factor loadings, in a (forced) four-factor model. The scree plot and parallel analysis supported a six-factor model: however, based on cross-factor loadings, homogeneity, and qualitative findings, we chose a four-factor model and named them based on the type of food items that loaded together: (1) ‘Sweets and sugary items’ because a majority of the food items included energy-dense, nutrient-poor food items except for fish and refrigerator access (7 items: frequency of soda, juice, and cookies purchase, buying juice, soda, and expensive types of fish, and having refrigerator access), (2) ‘Less preferred’, including primarily less preferred staple and cheaper sources of meat, and these households tended to receive foods as gifts (8 items: frequency of yucca, egg, and organ meat purchase, buying organ meat, yucca and canned tuna purchase, receiving and giving food), (3) ‘More preferred’ (5 items: not buying canned tuna, buying expensive fish, red meat, fish, and frequency of fish purchase), and (4) ‘Minimum meal,’ which was named because this included food items that constituted a base meal in this setting (5 items: purchase frequency of rice, onion, oil, plantains, and eggs). There were several variables with loadings higher than 0.3 on two factors (expensive fish, refrigerator access, frequency of egg purchase). Purchase of canned tuna negatively loaded in the ‘More preferred’ factor and positively loaded in the ‘Minimum meal’ factor, and was consistent with the discussions with the community field workers in that canned tuna is relatively cheaper than fresh fish or meat.

**Table 4:** Factor loadings of 22 items in the four-factor model for the Peruvian Amazon

Variables	Factor 1 “Sweet and sugary items”	Factor 2 “Less preferred”	Factor 3 “More preferred”	Factor 4 “Minimum meal”
Rice ^a^				0.8340
Onion ^a^				0.6423
Oil ^a^				0.8382
Plantains ^a^				0.4438
Yucca ^a^		0.7042		
Eggs ^a^		0.3850		0.4917
Organ meat ^a^		0.6917		
Organ meat		0.7216		
Canned tuna ^b^		0.4049	−0.3859	
Yucca ^b^		0.6986		
*Bujurqui* fish ^b^	0.3417		0.5055	
Red meat			0.4106	
Any fish ^a^			0.8078	
Any fish ^b^			0.8769	
Soda ^a^	0.5730			
Juice ^a^	0.8661			
Cookies ^a^	0.7620			
Juice ^b^	0.8421			
Cookies ^b^	0.8136			
Refrigerator access	0.3543		0.4789	
Receive gifted foods		0.6054		
Give or gift food		0.4444		

^a^Standardized frequency of purchase was used for these variables

^b^Purchase of the food item was used for these variables (yes/no)

##### Convergent validity (22 item, 4 factor model)

Table 5 shows bivariate correlations between the WAMI index (Water and sanitation, Assets, Maternal education, and Income composite) and HFIAS constructs with household FS by factors. Because the HFIAS and WAMI index (SES) survey were collected semiannually, this analysis only included a subset of the new FS survey collected in that same month, and in addition, there were seven SES forms missing at 36 months of age. The ‘Sweets and sugary items’ and ‘More preferred’ factors correlated with three components of the WAMI scale, particularly monthly income, maternal education, and modified assets, whereas the ‘Less preferred’ factor was negatively associated with all components of WAMI, especially assets. When compared to HFIAS status (none, mild, moderate, severe), the ‘Sweets and sugary items’ factor was negatively associated with overall HFIAS status, particularly with the quantity dimension. This indicates these purchase patterns of ‘Sweets and sugary items’ at the household level was negatively associated with worry over quantity of food. The ‘Less preferred’ and ‘Minimum meal’ factors were associated with quality components of the HFIAS tool.

**Table 5:** Convergent validity of the newly developed food security tool: Correlations between factors scores from the new tool with the existing household measures of socio economic status (components of the Water and sanitation, Assets, Maternal education, and Income composite (WAMI) index and of the Household Food Insecurity Access Scale)

	Correlation between sum of standardized household Factor Analysis Scores and household measures			
	“Sweet and sugar items” -- *mostly buying sweets, juices, expensive fish*	“Less preferred” -- *buying canned tuna, organ meat, yucca, eggs, receive/give food*	“More preferred” -- *red meat, fresh and expensive fish, refrigerator access*	“Minimum meal” -- *rice, yucca, plantains and egg*
WAMI	209 observations from 113 children (same month)			
Assets	0.22**	−0.19*	0.16*	−0.02
Monthly income (US$)	0.28***	−0.09	0.27***	0.12
Water/sanitation	0.15	−0.16*	0.17*	−0.05
Maternal Education	0.20**	−0.15	0.17*	0.06
HFIAS	215 observations from 113 children (same month)			
Overall FS status	−0.25**	0.16*	−0.04	0.17*
Quality	−0.01	0.29***	0.01	0.21**
Quantity	−0.24**	0.16	−0.13	0.11
Anxiety	−0.07	0.14	0.04	0.18*

Presented as mean correlation (*p* value). ^*^p < 0.10, ^**^p < 0.05, ^***^p < 0.01

##### Criterion related validity (22 item, 4 factor model)

Table 6 shows bivariate weighted (for repeat observations) correlations between the four factors and child nutrient intake and nutritional status. The ‘Sweet and sugary items’ factor had strong and positive correlations with a child’s intake of fats, animal source protein, fiber, and number of foods with desserts, grain, eggs, or meat. Similarly, the ‘More preferred’ category was associated with the same intake and food items except for fiber, grain and eggs. The number of food items with fish was negatively correlated with three groups, excluding the ‘Less preferred’ group, and in addition this was the only group that was associated with child’s intake of meat/fish protein (in grams). All three FAS groups except ‘Minimum meal’ were associated with child’s intake of food items with meat. Regarding the ‘Minimum meal’ group, there was an overall negative correlation with child’s intake of energy, fats, vitamin A, zinc, iron, animal source protein, and meat/fish protein intake. Micronutrient intakes were not associated with any of the factors. Only two groups − ‘Sweet and sugary items’ and ‘More preferred’ − had positive associations with dietary diversity. Correlation analysis of child weight for age (WAZ), length for age (LAZ), weight for length (WLZ), height for age (HAZ), and weight for height (WHZ) z scores with the four FS factor scores is shown in Table 6. Only the ‘More preferred’ group was positively and significantly associated with WAZ (*p*-value = 0.03) and WHZ (p-value = 0.05).

**Table 6:** Criterion validity of the newly developed food security tool: Correlations between factors scores from the new tool with child dietary intakes, and anthropometric status

	Correlation between sum of standardized household Factor Analysis Scores and children’s nutritional measures			
	“Sweet and sugary items” -- *mostly buying sweets, juices, expensive fish*	“Less preferred” -- *buying canned tuna, organ meat, yucca, eggs, receive/give food*	“More preferred” -- *red meat, fresh and expensive fish, refrigerator access*	“Minimum meal” -- *rice, yucca, plantains and egg*
Dietary intakes	922 child-days from 122 children			
Energy (kcal)	0.13	0.16*	0.12	−0.04
Fats (g)	0.29***	0.00	0.33***	−0.08
Animal source protein (g)	0.19**	0.07	0.25***	−0.20**
Meat/fish protein (g)	0.10	0.24***	0.04	−0.18*
Vitamin A(μg)	0.01	0.03	0.11	−0.04
Zinc (mg)	0.09	0.11	0.13	−0.07
Iron (mg)	0.06	−0.08	0.11	−0.15*
Sugar (g)	0.09	0.15*	0.06	−0.01
Fiber (g)	0.48***	0.23**	0.09	0.09
# Desserts/sweets	0.31***	0.09	0.22**	0.15
# Fish	−0.23**	0.07	−0.25***	−0.18*
# Grain	0.18*	0.25***	−0.01	0.18**
# Eggs	0.21**	−0.03	0.16*	0.04
# Meat	0.47***	0.18**	0.28***	0.07
Dietary diversity	0.50***	−0.03	0.20**	0.12
Nutritional status	997 observations from 116 children (826 observations from 76 children among 24 months+)			
WAZ	0.10	−0.05	0.19**	0.15
LAZ	−0.15	0.07	0.13	0.13
WLZ	0.04	0.25	0.05	0.26
HAZ	0.07	−0.16	0.14	0.13
WHZ	0.11	0.01	0.18*	0.09

Presented in correlation (p value) above. ^*^p < 0.10, ^**^p < 0.05, ^***^p < 0.01

#### Reliability (22 items, 4 factor model)

Reliability of the survey items was evaluated through Cronbach’s α, which examines how the items are related to each other, where a higher Cronbach’s α is indicative of higher internal consistency of survey items (DeVellis 2016). Food purchase (20 items) had a Cronbach’s α of 0.71 and frequency of purchase had an α of 0.81. Internal consistency of the 22-item scale had a Cronbach’s α of 0.73 (*n* = 181) and it also had relatively fair-to-good reliability when examined over 15 months, with Cronbach’s α ranging from 0.67 to 0.79.

## Discussion

We developed and validated a context-specific, experience-based food security (FS) measure for use in the Peruvian Amazon based on inputs from a team of community field workers (CFW), a literature review, and formative research. The development of the tool was nested within the MAL-ED birth cohort study to better capture the chronic and transitory FS experience in this community. Further, we aimed to develop a tool that captured all or most elements of the food insecurity experience in the Peruvian Amazon. In this community, household food security can be primary classified as phases 1A-3 based on FAO’s Integrated Food Security classification, where members vacillate between food secure to acute food insecurity during times of seasonal flooding (IPC Global Partners 2008).

The FGD with the CFWs shed light on several practices reflecting a household’s capacity to manage food insecurity. First, social ties in the community ameliorated the FS experience, which is supported by the factor loadings on the ‘Less preferred’ group, where gifting and receiving food loaded positively. This phenomenon has been observed in other parts of Peru (Sherman et al. 2016; Argumedo and Pimbert 2010). In the Andean region of Peru, Quechua communities widely practice *ayni*, a coping strategy based on reciprocity among neighbors, relatives and other socially obligated entities when resources are lacking (for work, food, goods) (Argumedo and Pimbert 2010). Here, barter markets are still a prominent way of procuring household goods and food in exchange for services such as childcare, housecleaning, and clearing land (Argumedo and Pimbert 2010). In urban Burkina Faso, Becquey and colleagues similarly found that adults’ social networks − defined as number of close friends and family members − positively influenced FS status during a food price crisis (Becquey et al. 2011).

Second, our findings indicate that the substitution of food with less preferred and less expensive staples and meat is a common occurrence during periods of food insecurity. For example, households substituted rice with yucca or plantains, and substituted fresh fish with canned tuna or organ meat. This phenomenon has been documented in other settings, where cassava was substituted for rice, and tofu for meat (Studdert et al. 2001). Further, when we summed food purchase frequency scores from the 35 pilot surveys based on food items from factor analysis for the ‘Less preferred’ group, and compared it to the pile-sorted categories, we found significant (Spearman rank) correlation with food security status as determined by the CFWs (−0.42, *P* < 0.01).

Finally, we learned that credit is differentially available to members of the communities. Specifically, we learned that having a stable (rather than seasonal) occupation and remittances was critical in determining the credit available to the household. Although it was beyond the scope of this study, factors constituting individuals’ accountability and ability to gain credit in this community, along with the role of social networks in ameliorating the FS experience, are important areas of research.

Overall, there was consistency of themes identified in the FGD and the factor analysis, which were further supported by associations with SES, HFIAS, dietary intake, and anthropometry. A key finding from the factor analysis revealed there were food purchase patterns that were distinctive to quality and quantity aspects of the HFIAS scale. Households that were deemed food insecure by the HFIAS scale purchased fewer sweets, and sugary items, and greater amounts of canned tuna and organ meat. Purchasing of items in the ‘Minimum meal’ and ‘Less preferred’ groups was also positively associated with worries over the quality of the food but not the quantity, suggesting that purchasing patterns and coping strategies such as receiving foods from relatives/neighbors are maintaining the sufficient quantity but not the preferred quality of food. Taken together, we have identified purchase patterns that are associated with different dimensions of the HFIAS scale.

We did not detect any associations between ‘Sweets and sugary items’ purchase patterns with the sugar consumption of children. This could have occurred for several reasons: First, we asked about purchase patterns at the household level, making it possible that children are not consuming some of these food items. Second, the household purchase patterns asked about the previous seven days, whereas dietary recalls assessed intake the day prior to interview. Third, based on dietary recalls data, we know that the most significant amounts of sugar are consumed by children in the form of homemade juices (‘*refrescos*’). Also, we did detect significant positive correlations of household purchase patterns of sweet and sugary items on a number of sweets and desserts consumed by the child. Although ‘Sweets and sugary items’ patterns were associated positively with animal source foods and fat intake, it was also associated with foods that were energy-dense and nutrient-poor such as desserts and cookies.

Criterion related validity revealed a high degree of specificity between the factors and nutrient intakes consumed by the child. For example, the purchase of sweets and juices was positively and significantly associated with the consumption of foods including sweets, fish, grains, eggs, and meat. The purchase of ‘More preferred’ foods was also positively correlated with child’s fat intake, animal source protein intake, and the consumption of food items with fish and meat − but not with other food items. We found unexpected negative associations of fish intake with two FAS groups. A likely explanation for these associations is that the definition of fish intake included canned seafood, thus the negative association with ‘Sweet and sugary items’ and ‘More preferred’ FAS because these groups included the purchase of fresh fish. Another unexpected finding was the lack of correlation between ‘More preferred’ with HFIAS domains and status. We posit that there may be two types of food secure households emerging in this community – one with preferences to purchase snacks and sugary beverages (‘Sweet and sugar items’), and the other with preferences to buy higher priced meat (red meat, expensive fish). The first pattern was not associated with child nutritional status, whereas the latter was positively associated with weight for age and weight for height measures.

There are several limitations to this study in that we lacked some key information, which would have enhanced the analysis and the conclusions we can draw. A household food inventory would have allowed us to evaluate the validity of the food purchase responses, as well as information on the quantity of food purchased. Second, we made no attempt to compare recent food expenditure (yesterday) to monthly food expenditure. Anecdotally, we do know that a household’s livelihood is structured from day-to-day in this community, and food expenditure in the previous day is reflective of that household’s purchasing power on that day.

There are many strengths to this study. First, the availability of concurrent tools such as the household level SES survey and HFIAS tool, repeated measures of dietary intake, and anthropometry for each child. Second, the involvement of community field workers, who served as a novel resource for the development and validation of the FS measure. Third, iterative pilot testing with the CFWs and the community for content, language, and interpretation of the survey. Lastly, the specificity of correlation of factor scores with the validity measures.

To our knowledge, this is the first experience-based FS tool developed in Peru. Globally, very few studies have been conducted on the relationship between food purchase frequency and type of food purchased with household food insecurity (Melgar-Quinonez et al. 2006; Rose and Charlton 2002). Two recent validation studies of the Food Access Survey Tool (FAST) in Bangladesh and Zambia revelated that rice and maize purchases frequency reflected the intensity of a coping strategy for food insecurity (Na et al. 2015; Na et al. 2016). For example, mild food insecurity led to more frequent purchases of smaller quantities of rice, but more severe food insecurity led to a high frequency of substitution of other staples for rice (Na et al. 2015). Another study conducted in the U.S. on food purchase patterns found that low-income households bought less expensive meat, fruits, and vegetables, but also smaller packages of cereals than high-income households (Kaufman et al. 1997). Becquey et al. (2011) reported similar findings in urban Burkina Faso, where in lean seasons, food insecure households relied more heavily on ready-to-eat foods such as packaged groundnut sauce. We see a similar phenomenon in that purchase of ready-to-eat canned tuna is associated with lower socio-economic status and is consumed more frequently when river levels are high, which is indicative of reduced fish availability and reduced access to forest products.

Utilizing food purchase and frequency and asking about access to a refrigerator is an objective, non-intrusive way of capturing household food security status and dietary patterns of households with young children in this community. Food purchase and frequency measures could be used as an indicator for acute changes in household food security status during specific phenomena (food price volatility, climate variability) with appropriate pilot testing and validation. Particularly, food purchase and frequency, and context specific behaviors at the household level can be used as surrogates for dietary intake patterns in children, and ultimately identify those at higher risk of poor nutritional status.

## References

[CR1] Argumedo A, Pimbert M (2010). Bypassing globalization: Barter markets as a new indigenous economy in Peru. Development.

[CR2] Ayllon, R. L. (2002). Evaluation of the use and management of fish resources in the pachitea river basin, Peruvian Amazon. FIU Electronic Theses and Dissertations. 1352.

[CR3] Becquey E, Delpeuch F, Konaté AM, Delsol H, Lange M, Zoungrana M, Martin-Prével Y (2011). Seasonality of the dietary dimension of household food security in urban Burkina Faso. British Journal of Nutrition.

[CR4] Bland JM, Altman DG (1995). Statistics notes: Calculating correlation coefficients with repeated observations: Part 2--correlation between subjects. British Medical Journal.

[CR5] Caulfield LE, Bose A, Chandyo RK, Nesamvuni C, De Moraes ML, Turab A (2014). Infant feeding practices, dietary adequacy, and micronutrient status measures in the MAL-ED study. Clinical Infectious Diseases.

[CR6] Chaparro MP, Estrada L (2012). Mapping the nutrition transition in Peru: Evidence for decentralized nutrition policies. Revista Panamericana de Salud Pública.

[CR7] Chung K (1997). Identifying the food insecure: The application of mixed-method approaches in India.

[CR8] Chuquiyauri R, Paredes M, Peñataro P, Torres S, Marin S, Tenorio A (2012). Socio-demographics and the development of malaria elimination strategies in the low transmission setting. Acta Tropica.

[CR9] Coates J, Wilde PE, Webb P, Rogers BL, Houser RF (2006). Comparison of a qualitative and a quantitative approach to developing a household food insecurity scale for Bangladesh. The Journal of Nutrition.

[CR10] DeVellis, R. F. (2016). *Scale development: Theory and applications*. Applied Social Research Methods, vol. 26. Los Angeles: SAGE Publishing.

[CR11] Food and Agriculture Organization (1996). Rome declaration on world food security and world food summit plan of action: World food summit 13–17 November 1996.

[CR12] Frongillo EA (1999). Validation of measures of food insecurity and hunger. The Journal of Nutrition.

[CR13] Frongillo EA, Nanama S (2006). Development and validation of an experience-based measure of household food insecurity within and across seasons in northern Burkina Faso. The Journal of Nutrition.

[CR14] Frongillo EA, Chowdhury N, Ekström E-C, Naved RT (2003). Understanding the experience of household food insecurity in rural Bangladesh leads to a measure different from that used in other countries. The Journal of Nutrition.

[CR15] Gajate G, Inurritegui M (2003). El impacto del Vaso de Leche sobre el nivel de nutrición infantil. Economía y Sociedad.

[CR16] Gajate-Garrido G (2013). Excluding the rural population: The impact of public expenditure on child malnutrition in Peru. The World Bank Economic Review.

[CR17] Gittelsohn J, Mookherji S, Pelto G (1998). Operationalizing household food security in rural Nepal. Food and Nutrition Bulletin.

[CR18] Haddad L, Sullivan J, Kennedy E (1992). Identification and evaluation of alternative indicators of food and nutrition security: Some conceptual issues and an analysis of extant data. Food and nutrition monitoring project.

[CR19] Instituto Nacional de Estadística e Informática (Perú) (2015). Perú: encuesta demográfica y de salud familiar ENDES 2014.

[CR20] IPC Global Partners (2008). Integrated Food Security Phase Classification Technical Manual. Version 1.1..

[CR21] Jones AD, Ngure FM, Pelto G, Young SL (2013). What are we assessing when we measure food security? A compendium and review of current metrics. Advances in Nutrition.

[CR22] Kaufman PR, MacDonald JM, Lutz SM, Smallwood DM (1997). Do the poor pay more for food? Item selection and price differences affect low-income household food costs.

[CR23] Leroy JL, Ruel M, Frongillo EA, Harris J, Ballard TJ (2015). Measuring the food access dimension of food security: A critical review and mapping of indicators. Food and Nutrition Bulletin.

[CR24] Limon G, Fournié G, Lewis EG, Dominguez-Salas P, Leyton-Michovich D, Gonzales-Gustavson EA (2017). Using mixed methods to assess food security and coping strategies: A case study among smallholders in the Andean region. Food Security.

[CR25] Lorenzana PA, Sanjur D (1999). Abbreviated measures of food sufficiency validly estimate the food security level of poor households: Measuring household food security. The Journal of Nutrition.

[CR26] MAL-ED Network Investigators (2014). The MAL-ED study: A multinational and multidisciplinary approach to understand the relationship between enteric pathogens, malnutrition, gut physiology, physical growth, cognitive development, and immune responses in infants and children up to 2 years of age in resource-poor environments. Clinical Infectious Diseases.

[CR27] Maxwell DG (1996). Measuring food insecurity: the frequency and severity of “coping strategies”. Food Policy.

[CR28] Maxwell D, Ahiadeke C, Levin C, Armar-Klemesu M, Zakariah S, Lamptey GM (1999). Alternative food-security indicators: Revisiting the frequency and severity of `coping strategies. Food Policy.

[CR29] Melgar-Quinonez HR, Zubieta AC, MkNelly B, Nteziyaremye A, Gerardo MFD, Dunford C (2006). Household food insecurity and food expenditure in Bolivia, Burkina Faso, and the Philippines. The Journal of Nutrition.

[CR30] Na, M., Gross, A. L., & West, K. P. (2015). Validation of the food access survey tool to assess household food insecurity in rural Bangladesh. *BMC Public Health*, 1–10. 10.1186/s12889-015-2208-1 PMC4561472 26346311

[CR31] Na M, Gross AL, Wu LS-F, Caswell BL, Talegawkar SA, Palmer AC (2016). Internal validity of the food access survey tool in assessing household food insecurity in rural Zambia. Food Security.

[CR32] Pérez-Escamilla R (2012). Can experience-based household food security scales help improve food security governance?. Global Food Security.

[CR33] Psaki SR, Seidman JC, Miller M, Gottlieb M, Bhutta ZA, Ahmed T (2014). Measuring socioeconomic status in multicountry studies: Results from the eight-country MAL-ED study. Population Health Metrics.

[CR34] Rose D, Charlton KE (2002). Quantitative indicators from a food expenditure survey can be used to target the food insecure in South Africa. The Journal of Nutrition.

[CR35] Ryan GW, Bernard HR (2016). Techniques to identify themes. Field Methods.

[CR36] Sherman M, Ford J, Llanos-Cuentas A, Valdivia MJ, Bussalleu A (2015). Vulnerability and adaptive capacity of community food systems in the Peruvian Amazon: A case study from Panaillo. Natural Hazards.

[CR37] Sherman M, Ford J, Llanos-Cuentas A, Valdivia MJ (2016). Food system vulnerability amidst the extreme 2010–2011 flooding in the Peruvian Amazon: A case study from the Ucayali region. Food Security.

[CR38] StataCorp (2013). Stata statistical software: Release 13.

[CR39] Stifel D, Alderman H (2006). The “Glass of Milk” subsidy program and malnutrition in Peru. The World Bank Economic Review.

[CR40] Studdert LJ, Frongillo EAJ, Valois P (2001). Household food insecurity was prevalent in Java during Indonesia's economic crisis. The Journal of Nutrition.

[CR41] Swierk L, Madigosky SR (2014). Environmental perceptions and resource use in rural communities of the Peruvian Amazon (Iquitos and vicinity, Maynas Province). Tropical Conservation Science.

[CR42] Swindale A, Bilinsky P (2006). Development of a universally applicable household food insecurity measurement tool: Process, current status, and outstanding issues. The Journal of Nutrition.

[CR43] Vargas S, Penny ME (2009). Measuring food insecurity and hunger in Peru: A qualitative and quantitative analysis of an adapted version of the USDA’s food insecurity and hunger module. Public Health Nutrition.

[CR44] World Health Organization (WHO) (2010). *Indicators for assessing infant and young child feeding practices: part 2: measurement*. Geneva: WHO.

[CR45] Yori PP, Lee G, Olortegui MP, Chavez CB, Flores JT, Vasquez AO (2014). Santa Clara de Nanay: The MAL-ED cohort in Peru. The Journal is Clinical Infectious Diseases.

